# Effect of hCG priming on embryonic development of immature oocytes collected from unstimulated women with polycystic ovarian syndrome

**DOI:** 10.1186/1477-7827-10-40

**Published:** 2012-05-24

**Authors:** Xiaoying Zheng, Lina Wang, Xiumei Zhen, Ying Lian, Ping Liu, Jie Qiao

**Affiliations:** 1 Department of Obstetrics and Gynecology, Center of Reproductive Medicine, Peking University, Third Hospital, Beijing, 100191, China; 2 Key Laboratory of Assisted Reproduction, Ministry of Education, Beijing, 100191, China

**Keywords:** Polycystic ovarian syndrome, *In vitro* maturation, hCG priming

## Abstract

**Backgroud:**

The effect of hCG priming on oocyte maturation and subsequently outcome in IVM cycles has remained a debated issue. A randomized controlled study was performed to investigate whether or not hCG priming prior to oocyte aspiration can improve the developmental competence of immature oocytes from unstimulated ovaries in women with polycystic ovarian syndrome (PCOS).

**Methods:**

Eighty two patients with PCOS underwent IVM cycles. Each patient was randomly assigned to the hCG-primed (10,000 IU) or non-primed groups 36–38 hours before oocyte retrieval depending on the computerized random table. After the oocytes had *in vitro* matured, fertilization, culture and embryo transfer were performed.

**Results:**

The average number of *cumulus*-oocyte complexes (COCs) recovered was 13.80 and 14.35 in the hCG-primed and non-primed groups, respectively (*p* > 0.05). The maturation rate of COCs was significantly improved in the hCG-primed group (55.43% vs. 42.29%; *p* < 0.05). The fertilization and cleavage rates were comparable between the groups. The hCG-primed and non-primed groups did not differ with respect to the clinical pregnancy (37.50% vs. 50.00%), live birth (22.50% vs. 30.95%), and implantation rates (32.86% vs. 32.56%). The pregnancy losses was 6 (40.00%) of 15 clinical pregnancies in the hCG-primed group, and 8 (38.10%) of 21 clinical pregnancies in the non-primed group.

**Conclusions:**

While a significant improvement in the nuclear maturation rate of immature oocytes was observed in hCG-primed IVM cycles with PCOS patients, the use of hCG prior to oocyte retrieval did not improve the subsequent embryo developmental competence. The high rate of pregnancy loss in IVM cycles should receive more attention.

## Background

Polycystic ovarian syndrome (PCOS) is a common endocrine disorder characterized by ovarian hyperandrogenism, insulin resistance, and paracrine dysregulation of follicle development [1,2]. PCOS has a negative effect on female fecundity. The estimated prevalence of PCOS is 5%–10%, as reported in population-based studies [3]. Trounson *et al.*[4] described the first pregnancy and delivery of a healthy baby after *in vitro* maturation (IVM) of immature oocytes obtained from a patient with PCOS. Over the next decade, immature oocyte retrieval followed by IVM has become a widespread treatment for infertile women with PCOS because there are numerous antral follicles within the ovaries in this group of patients. Compared with ovary-stimulated *in vitro* fertilization (IVF), the major advantages of IVM include avoidance of the risk of ovarian hyperstimulation syndrome, reduced cost, and simplification of treatment. Nevertheless, IVM has not been adopted as a mainstream method in infertility treatment even though reasonable results have been reported by some clinics [5-7]. The explanation for this is the fact that IVM tends to have a lower rate of live births per treatment compared with conventional IVF. A number of factors might lead to the lower live births rate of IVM, including non-synchronization of oocyte nuclear and cytoplasmic maturation, suboptimal culture conditions, an endocrine disturbance and suboptimal timing of insemination [8-11].

Gonadotropins play an important role in the regulation of oocyte growth and maturation. In order to mimic the pre-ovulatory luteinizing hormone (LH) surge in spontaneous menstrual cycles, human chorionic gonadotropin (hCG) is routinely administered at a dose of 5000–10000 IU as a surrogate for LH at the end of follicular stimulation to trigger the resumption of meiosis and nuclear maturation of oocytes using IVF technology. The traditional application of hCG has shown to be highly successful and valuable tools in the treatment of infertility for > 4 decades [12]. However, the effect of hCG priming on oocyte maturation and developmental competence in IVM cycles has remained a contested issue. Chian and colleagues [13] demonstrated that hCG priming could speed up the maturation time of oocytes in women with PCOS. Subsequently, the results of a multicentre study by the same investigators provided further support for this initial finding by reporting pregnancy rates of 30%–35% in hCG-treated IVM cycles in patients with PCO and PCOS [5]. However, similar studies did not demonstrate a beneficial effect of hCG priming [6,14]. Among the many reports [5,6,9-11,13,14] involving pregnancy rates after IVM with gonadotropin priming, there is limited data generated from randomized controlled clinical studies [13]. Therefore, the present study was designed according to a randomized controlled paradigm to demonstrate whether or not hCG priming prior to oocyte aspiration involving IVM oocytes from unstimulated women with PCOS can improve embryonic developmental competence and yield respective favorable clinical outcomes.

## Methods

### Patients

This protocol was approved by the Ethics Committee of the Peking University (the registration number: 2006FC001). Between January 2007 and December 2008, 82 patients with PCOS underwent IVM cycles at the Reproductive Centre of Peking University Third Hospital. These patients consistently met the Rotterdam ESHRE/ASRM consensus criteria for the diagnosis of PCOS [1]. The mean age of the patients was 30.2 years (range, 24–39 years). All patients had oligo-anovulation and presented with irregular menstrual cycles (35–90 days) or amenorrhoea. Written informed consent was obtained from each patient before undergoing treatment.

### *In vitro* maturation protocol

To initiate the IVM treatment cycle in anovulatory patients, the patients received intramuscular injections of progesterone (Progesterone Injection; Xianju Pharmacy, Zhejiang, China) 40 mg daily for 7 days. A withdrawal bleed occurred within 7 days after the last dose. A baseline ultrasound scan was obtained on day 2 or 3 following the onset of menstrual bleeding to ensure that no ovarian cysts were present. For the purpose of the current study, women were randomly allocated to one of two groups depending on the computerized random table on the day of the first baseline ultrasound scan, as follows: primed with 10,000 IU hCG; or no priming. The patients and the embryologist were blind to the random results. Transvaginal ultrasound scans were repeated on days 6–8 to exclude the development of a dominant follicle. Oocyte retrieval was scheduled once the endometrium had reached at least 6 mm in thickness and there was no follicle larger than 10 mm. According the random results, the hCG (Profasi, 10000 IU, Serono, Aubonne, Switzerland) was given subcutaneously to patients in the hCG-primed group. Immature oocyte collection was performed 36–38 h after hCG priming; while oocyte retrieval was undertaken directly in the non-primed group without hCG injection.

Transvaginal ultrasound–guided collection of oocytes was performed with a specially designed 19 G single-lumen aspiration needle (K-OPS-7035-REH-ET; Cook, Queensland, Australia). For aspiration, the suction pressure was reduced to 80 mm Hg. Follicular aspirates were collected in 10 mL culture tubes and filtered through a cell strainer (Cell Strainer 352350, 70 μm nylon; Falcon, MA, USA). After filtering, the collected aspirates were rinsed with pre-warmed DPBS (Sigma-Aldrich, St. Louis, MO, USA) and transferred to a 100x15 mm Petri dish (Nunc, Roskilde, Denmark) to search for *cumulus*-oocyte complexes (COCs) under a stereomicroscope. All COCs were then transferred into IVM oocyte medium (IVM media kit; Sage, CT, USA) supplemented with 0.075 IU/mL FSH and 0.075 IU/mL LH (Menopur; Ferring, Kiel, Germany) for maturation in 5% CO_2_ incubator at 37°C with satured humidty. After 28–32 h of culture, the oocytes were denuded of *cumulus* cells for observing the presence of a first polar body extrusion. All the metaphase II (MII) oocytes were inseminated by means of intracytoplasmic sperm injection (ICSI).

Fertilization was considered normal when two pronuclei were present between 16 and 18 h after ICSI. Only normally fertilized oocytes were further considered for embryo transfer or cryopreservation. All zygotes were cultured in cleavage medium (G-M, LifeGlobal, CT, USA) supplemented with 10% synthetic serum substitute (SSS; Irvine Scientific, Santa Ana, CA, USA) up to day 3 after ICSI. Prior to transfer or cryopreservation, embryonic development was assessed according to the developmental stage and degree of cytoplasmic fragmentation. Two or three of the morphologically best embryos were selected for fresh transfer and supernumerary good-quality embryos (4–8 cells, ≤30% fragmentation, even size) were cryopreserved.

### Endometrial preparation and luteal support

For preparation of the endometrium, patients were given oestradiol valerate (Progynova, 6 mg orally; Schering, Berlin, Germany) starting on the day of oocyte retrieval. Luteal support was provided with 60 mg of progesterone (Progesterone Injection, Xianju Pharmacy, Zhejiang, China) intramuscularly daily starting from the day of ICSI. Both medications were continued until either a pregnancy test was negative or a positive fetal heartbeat was observed.

The serum hCG concentration was determined 14 days after embryo transfer (ET) and again 1 week later. Clinical pregnancy was defined as the presence of an intrauterine gestational sac on ultrasound examination on day 35 after ET. The implantation rate was calculated as the number of gestational sacs identified on ultrasound per number of transferred embryos.

### Statistical analysis

Statistical analysis was performed using SPSS 10.0. Differences were considered significant at *p* < 0.05. For comparison of the mean variables, Student’s *t-*test was used. A χ^2^ analysis was used between group comparisons of reproductive outcomes, including implantation, clinical pregnancy, and birth rates.

## Results

Basic patient characteristics, including mean female age, body mass index (BMI), and baseline FSH did not differ between the hCG-primed (40 women) and the non-primed groups (42 women; Table 1).

**Table 1:** Characteristics of the PCOS patients receiving IVM treatment

	**hCG-primed group**	**Non-primed group**	**p**
*Patients*	40	42	
*age* (years)	30.03 ± 3.65	30.33 ± 3.71	ns
*BMI* (kg/m^2^)	24.93 ± 4.94	25.37 ± 5.75	ns
*No. of primary infertility * (%)	34 (85.00)	37 (88.10)	ns
*Basal FSH* (IU/mL)	5.57 ± 1.76	6.08 ± 1.66	ns
*Antral follicle count*	22.60 ± 5.93	22.76 ± 5.99	ns
*No. of anovulatory* (%)	36 (90.00)	34 (80.95)	ns
*No. of patients with biochemical hyperandrogenism* (%)	25 (62.5)	20 (47.62)	ns

**Figure 1:**
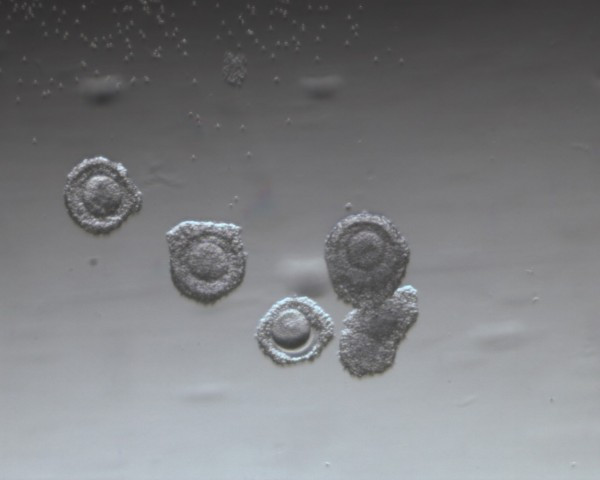
Immature oocytes with compacted or sparse cumulus in the non hCG-primed group.

**Figure 2:**
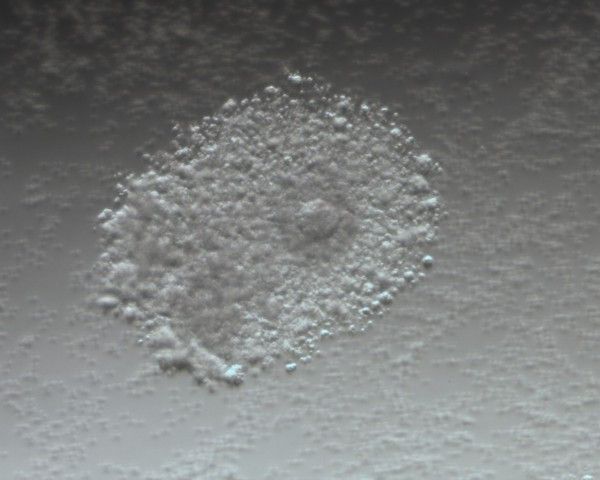
Immature oocytes with dispersed cumulus in the hCG-primed group.

The average number of COCs recovered was 13.80 and 14.35 in the hCG-primed and non-primed groups, respectively (*p* > 0.05). However, the COCs had a different appearance between the two groups. All COCs in the non-primed group had a compacted or sparse cumulus (Figure 1), while nearly 35% of immature oocytes in the hCG-primed group were with a dispersed cumulus (Figure 2). The maturation rate of COCs was significantly improved in the hCG-primed group (55.43% vs. 42.29%; *p* < 0.05). However, the fertilization and cleavage rates were comparable between the hCG-primed and non-primed groups (Table 2).

**Table 2:** ***In vitro *maturation outcome of PCOS women with or without hCG-priming**

	**hCG-primed group**	**Non-primed group**	**p**
*No. of oocyte retrieval cycles*	40	42	
*No. of oocytes retrieved*			
*Total (range)*	552 (1–50)	603(1–47)	
*Mean*	13.80	14.35	ns
*No. of oocytes matured at 32 h*			
*Total (range)*	306 (1–21)	255 (0–18)	
*Mean*	7.65	6.07	
*Maturation rate (%)*	55.43	42.29	0.000
*No. of oocytes fertilized (%)*	194 (63.40)	167 (65.49)	ns
*No. of oocytes cleaved (%)*	192 (98.97)	166 (99.10)	ns
*Cycles with embryo transfer (%)*	34 (85.0)	40 (95.24)	ns
*No. of embryos transferred (mean)*	70 (2.06)	86 (2.15)	ns
*No. of clinical pregnancies*	15	21	
*Clinical pregnancy rate per OR*^*a*^*(%)*	37.50	50.00	ns
*Clinical pregnancy rate per ET*^*b*^*(%)*	44.12	52.50	ns
*No. of embryos implantation (%)*	23 (32.86)	28 (32.56)	ns
*No. of live birth (% per OR)*	9 (22.50)	13 (30.95)	ns
*Singleton*	5	11	
*Twins*	4	2	
*No. of infants*	13	15	
*No. of miscarriage (%)*^*c*^	6 (40.00)	8 (38.10)	ns
*Early miscarriage (<12 weeks)*	3	5	
*Late miscarriage (12–28 weeks)*^*d*^	3	3	
*No. of cycles with embryo freezing*	19 (47.50)	13 (30.95)	ns

^a^*OR* oocyte retrieval.

^b^*ET* embryo transfer.

^c^ Miscarriage was defined as pregnancy loss after ultrasonographic evidence of an intrauterine pregnancy.

^d^ Late miscarriage was defined as pregnancy loss between 12 and 28 weeks of gestation, which includes any termination of pregnancy after diagnosis of a congenital abnormality.

Good-quality embryos (4–8 cells, ≤30% fragmentation) were transferred on day 3 after ICSI. Embryo transfers were cancelled for 6 cases in the hCG-primed group because of a failure of fertilization (n = 3) or poor embryo development (n = 3). For 2 cases in the non-primed group, transfers did not take place because of a lack of matured oocytes acquired in 32 h (n = 1) or poor embryo quality (n = 1). The details of the pregnancy outcomes are shown in Table 2. There was no significant difference in the clinical pregnancy, live birth, and implantation rates between the two groups. The number of late fetal losses was 3 in each group, including one termination for a congenital cardiac abnormality in the non-primed group and one for abnormal neurologic development in the hCG-primed group.

As of October 2010, fresh embryo transfers after IVM resulted in the births of 28 infants (15 boys and 13 girls) in 2 groups. There were 9 delivers and 13 babies (5 singletons and 4 twins) in hCG-primed group, meanwhile 13 delivers and 15 babies (11 singletons and 2 twins) in non-primed group. The details of the pregnancies are shown in Table 2. In addition, one singleton baby in the non-primed group was born through 16 frozen-thawed embryo transfers, including 10 cases in hCG-primed and 6 cases in non-primed group.

## Discussion

It is known that LH receptors are expressed by granulosa cells in the late stages of follicle development when antral ovarian follicle diameter increases beyond approximately 10 mm [15]. Therefore, queries about the hCG effect on the IVM cycles in which most follicle diameters were < 10 mm continue. The rationale for hCG priming in IVM cycles in women with PCOS was based on the finding that *in vivo* administration of hCG enhances the nuclear maturation of oocytes [13], although the underlying mechanism is unclear. Our randomized controlled study demonstrated also an improved maturation rate of COCs with hCG-priming. LH receptors in theca cells may be a partially explanation for that. It is well known that theca cells express LH receptors from the secondary follicle stage, and LH is capable of stimulating androgen substrate production. Androgens can be transformed into estrogens through the action of the aromatase system, and then estrogens in turn play a role in improving follicle and oocyte maturity. Another explanation may due to the prematurely expression of LH receptor in granulosa cells from polycystic ovaries [16]. Indeed, Yang *et al. *[17] observed LH receptor expression in cumulus cells of oocytes generated from IVM cycles of PCOS patients. Although the follicle diameter was less than 10 mm, the granulosa cells become receptive to hCG stimulation; hCG was then capable of exerting its actions, including initiating a cascade of molecular events, stimulation of steroid production [18] and extracellular matrix production, known as cumulus expansion or mucification [19] in the COCs. In fact, we observed that nearly one-third of COCs have dispersed cumulus cells in the hCG-primed group, while all COCs were compact or sparse cumulus cells in the non-hCG primed group. This is consistent with LH surge that, in vivo, induces the cumulus expansion. So on, the hCG treatment “found” some receptors to produces a mimetic action similar to LH surge.The immature oocytes with dispersed cumulus cells at the time of collection have a high nuclear maturation rate among PCOS patients [20]. Some reports indicated that hCG priming during IVM cycles in women without PCO and regular menstrual cycles had not been shown to have a beneficial effect with respect to the maturation rate of oocytes [6,11,14]. The discrepancy in the hCG priming effect on COCs maturation in IVM cycles may be explained by the selection differences amongst patients. The abnormally premature expression of LH receptor in granulosa cells was only found from polycystic ovaries [16], and then hCG can exert the action through the LH receptor.

Complete maturation of oocyte is essential for the developmental competence of embryos. Non-synchronization of oocyte nuclear and cytoplasmic maturation was considered to be a major reason for poor developmental competence of IVM oocytes [21,22]. hCG administration *in vivo* can fasten the nuclear maturation process, as discussed above, while the effect on cytoplasmic maturation was unclear. Cytoplasmic maturation was important in preparation for fertilization and early embryo development, the inadequacy tends to present later in development as impaired embryo cleavage or implantation failure. We found no substantial improvement in the developmental competence of oocytes in the current study, although hCG priming before oocyte retrieval promoted oocyte nuclear maturation. The clinical endpoints (pregnancy and live birth rates), of the primed and non-primed groups showed no significant differences. Similarly, an improved maturation rate was not reflected in the pregnancy outcomes in the report by Chian *et al.*[13], which favored hCG priming first. Anttila *et al.*[6] did not find a beneficial effect of hCG priming on the number of oocytes collected, and the fertilization or cleavage rates compared with non-primed patients undergoing IVM. The lack of effect of hCG priming on clinical indices could reflect that hCG priming was not enough to improve the cytoplasm maturation. Indeed, how to optimize the clinical regimen and embryo culture conditions to achieve the best synchronized oocyte nuclear and cytoplasmic maturation for IVM merits further research.

Another important reason which limited the widespread uptake of IVM was the question of safety, including pregnancy loss, congenital abnormality and post-natal outcomes. The miscarriage rate in IVM pregnancies varies between 25% and 57% [23]. This could be regarded as high, but conclusions are difficult to draw as the data come from observational studies without adequate controls. In our investigation, the miscarriage rate of IVM was nearly 40% and higher than conventional IVF/ICSI (15%) during the same time period in our clinic, whether with or without hCG priming. It was suggested that hCG priming was not useful to decrease pregnancy loss. The basis for high pregnancy loss of IVM in PCOS has yet to be investigated. Possible explanations include unknown effects from special patient population, poorer embryo quality, or suboptimal endometrial preparation. Some investigators have observed that the clinical miscarriage rate in IVM pregnancies is not higher than IVF pregnancies when controlled for patient characteristics [24,25]. Buckett *et al.*[24] proposed that a higher rate of pregnancy losses after IVM is related to PCOS rather than the IVM procedure itself. On the other hand, Confocal microscopic analysis of the spindle and chromosome structure of human IVM oocytes have shown that there is a higher frequency of abnormal meiotic spindle and chromosomal alignment than *in vivo* matured oocytes [26]. Additional studies are needed to clarify the roles of IVM and PCOS in the observed miscarriage cases to further our understanding in helping infertile women of PCOS undergoing IVM. It is worth noting that there was one case of pregnancy termination because of congenital malformations in each group. In our study, no congenital defect had been found in the 28 delivered babies at birth. Due to the very low numbers of IVM infants in present and other study, it is impossible at present to determine accurately whether IVM is associated with an increased risk of congenital birth defects. Careful long-term follow-up studies need to be conducted.

## Conclusions

In conclusion, this randomized controlled study showed a significant improvement in the nuclear maturation of immature oocytes in hCG-primed IVM cycles in PCOS patients, while the respective embryo competence improvement was not observed through the use of hCG prior to oocyte retrieval. Acceptable live birth results can be achieved in PCOS patients in the hCG-primed and non-primed groups. The high number of pregnancy losses registered in the present study call for caution in clinical practice with respect to IVM. hCG priming was not useful in decreasing the miscarriage rate. How to optimize the clinical regimen and embryo culture conditions to improve oocyte development competence needs further study.

## Competing interests

The authors declare that they have no competing interests.

## Authors’ contributions

All authors participated in the design, interpretation of the studies, analysis of the data and review of the manuscript; Prof. QJ and LP concept and designed the study. ZXY, WLN, ZXM, LY and LP conducted the experiments; ZXY and WLN wrote the manuscript. Prof. QJ revised the article critically for important intellectual content. All authors read and approved the final manuscript.
